# Construction of a sepsis recovery-failure axis and single-cell anchoring using public multi-cohort whole-blood and single-cell transcriptomic data

**DOI:** 10.3389/fmed.2026.1861140

**Published:** 2026-08-06

**Authors:** Ye Tan, Zhongyang Wang, Jinqiu Ding, Yongpeng Xie

**Affiliations:** 1 Lianyungang Clinical College of Nanjing Medical University, Lianyungang, Jiangsu, China; 2 The First Affiliated Hospital of Kangda College of Nanjing Medical University, Lianyungang, Jiangsu, China; 3 Department of Emergency Medicine, The First People’s Hospital of Lianyungang, Lianyungang, Jiangsu, China; 4 The Affiliated Lianyungang Hospital of Xuzhou Medical University, Lianyungang, Jiangsu, China

**Keywords:** bioinformatics, classical monocytes, host state, recovery-failure axis, sepsis, single-cell transcriptomics, translational medicine

## Abstract

Sepsis exhibits marked heterogeneity in host responses. Although prior time-course transcriptomic studies have provided important information about sepsis diagnosis and mortality-associated gene-expression dynamics, many bioinformatic signatures remain optimized for cross-sectional classification or endpoint labels. Using public multi-cohort whole-blood transcriptomic and single-cell datasets, we constructed a sepsis recovery-failure axis constrained by longitudinal trajectory rules and evaluated its cross-cohort transferability, clinical relevance, and single-cell anchoring. In GSE54514, we identified 20 recovery-related genes and 20 failure-related genes and defined a recovery-failure score. Survivors showed a non-monotonic but ultimately upward movement by day 5, whereas non-survivors progressively shifted toward the failure end of the axis, with clear separation by day 5. Linear mixed-effects models using all repeated measurements from day 1 to day 5 showed a significant interaction between time and outcome that remained stable after adjustment for neutrophil proportion and baseline APACHE II. GSE57065 and GSE65682 supported transfer of the axis for host-state stratification, while also showing that cohort-wise standardization limits interpretation as a universal absolute cutoff. Additional null and cross-cohort structure analyses showed that external-cohort patient-level score variance exceeded matched random-gene expectations and that high-score patients across external cohorts shared non-axis transcriptomic effects. The score showed only weak correlations with bedside severity scores, suggesting that it may serve as a complementary host-state indicator rather than a replacement for clinical severity scores. At the single-cell level, broad PBMC annotation followed by monocyte refinement localized the strongest donor-level axis signal to classical monocytes; additional T-cell cluster screening did not identify a robust hidden T-cell cluster that superseded monocyte anchoring. In non-survivors, classical monocytes exhibited reduced interferon responses and oxidative phosphorylation together with enhanced inflammatory, IL6-JAK-STAT3, and coagulation programs. Axis displacement within interferon-activated monocytes was supported by limited cellular coverage and should be treated as exploratory. Taken together, the recovery-failure axis is a longitudinally derived host-state score that may support sepsis state stratification, disease-course monitoring, and hypothesis generation, but ***requires prospective calibration before patient-level clinical use.***

## 1 Introduction

Sepsis is a life-threatening syndrome caused by a dysregulated host response to infection (1). Its pathophysiology does not reflect a single abnormal pathway; rather, multiple disturbed programs, including amplified inflammation (2), immunosuppression (3), coagulation imbalance (4), endothelial injury, and metabolic reprogramming (5), emerge in different proportions, orders, and intensities across patients. For this reason, sepsis has long been regarded as one of the most difficult clinical syndromes to explain and stratify using any single biomarker.

Previous sepsis transcriptomic studies have generated diagnostic signatures, co-expression modules, machine-learning features, molecular endotypes, and candidate hub molecules (6–8). Importantly, sepsis transcriptomics has not been restricted to single-time-point designs: earlier multicohort and machine-learning studies used time-course gene-expression data to identify robust diagnostic or temporally stable mortality-associated markers (9, 10). The present study therefore does not claim to be the first time-course analysis of sepsis. Rather, it addresses a narrower gap: many applied bioinformatic analyses still emphasize what is higher or lower at one contrast, or optimize a case-control or survival label, without explicitly defining a transferable state coordinate that links longitudinal recovery direction, external cohort transfer, and single-cell cellular anchoring.

When sepsis is reconsidered from the perspective of disease-course evolution, adverse outcome may not simply mean stronger inflammation. More plausibly, it may indicate failure of the host to move from an acutely dysregulated state into an orderly recovery trajectory. In other words, the key determinant of outcome may not be whether a specific inflammatory pathway is transiently higher, but whether recovery-related programs can be established in time, maintained stably, and ultimately suppress persistent injury programs (11). On this basis, we reasoned that a dynamic state axis could complement marker discovery by describing host recovery capacity and failure tendency.

Public databases provide a practical foundation for this strategy (12). GEO contains whole-blood transcriptomic cohorts with healthy controls, longitudinal time points, and clinical outcomes that can be used to define the direction of disease-course dynamics. At the same time, peripheral-blood single-cell datasets are available to project whole-blood-level states onto specific immune-cell programs. Integrating these two data types offers an opportunity to move sepsis bioinformatics beyond “biomarker screening” toward “state modeling and cellular anchoring.”

On this basis, we proposed the concept of a recovery-failure axis. In the discovery cohort, this axis was constrained by four forms of recovery evidence and two forms of failure evidence rather than derived from a single comparison. In external whole-blood cohorts, we tested its transferability and value for host-state stratification. We further asked whether the transferred heterogeneity exceeded random-gene expectations and whether high-score patients in independent cohorts shared transcriptome-wide structure beyond the score genes themselves. At the single-cell level, we sought to identify its principal cellular carrier, characterize intracellular program abnormalities, and assess differences in cross-cell communication. The central question was this: what kind of host state corresponds to poor outcome in sepsis, and can this state be primarily localized at the single-cell level to signals related to classical monocytes rather than to a masked T-cell subcluster signal?

## 2 Materials and methods

### 2.1 Study design and data sources

A total of five public cohorts were included. Whole-blood transcriptomic datasets comprised GSE54514 (13), GSE57065 (14), and GSE65682 (15). GSE54514 served as the discovery cohort and included 163 samples: 36 samples from 18 healthy controls, 96 longitudinal samples from 26 survivors, and 31 longitudinal samples from 10 non-survivors. This cohort was used to define the recovery-failure axis and analyze disease-course trajectories. GSE57065 contained 107 samples, including 25 healthy controls and 82 serial samples from 28 patients with septic shock, and was used for cross-cohort transfer validation. GSE65682 comprised 802 samples, including 42 healthy controls and 760 intensive care unit patients, of whom 479 also had 28-day outcome and MARS molecular endotype information, and was used to reinforce analyses of host-state heterogeneity.

Single-cell datasets included GSE167363 (16) and GSE151263 (17). GSE167363 was a mortality-related single-cell cohort with 12 samples; after quality control, 59,227 cells from seven donors were retained, including two healthy controls, three survivors, and two non-survivors, with sampling at 0 and 6 h. GSE151263 served as an external single-cell generalization cohort with seven samples; after quality control, 24,796 cells were retained from four patients with sepsis alone and three patients with sepsis complicated by acute respiratory distress syndrome.

### 2.2 Whole-blood transcriptomic preprocessing and differential analysis

After preprocessing, all whole-blood cohorts were unified at the gene-symbol level, duplicate probes were collapsed, and clinical groupings, time points, and subject identifiers were curated according to public sample annotations. Because GSE54514 included repeated measurements, differential analysis was performed within the limma framework (18) with duplicate correlation used to estimate within-subject correlation. Four prespecified contrasts were used for axis construction: survivors on day 5 versus day 1, non-survivors on day 5 versus day 1, survivors versus non-survivors on day 5, and survivors on day 1 versus healthy controls on day 1. In this manuscript, “survivor time-course log_2_ fold change (logFC)” refers specifically to the limma-estimated log_2_ fold change for survivor day 5 minus survivor day 1 in GSE54514; “non-survivor time-course logFC” is defined analogously as non-survivor day 5 minus non-survivor day 1.

### 2.3 Definition of the recovery-failure axis

Construction of the recovery-failure axis followed a prespecified biological logic. Recovery-related genes were required to satisfy four criteria simultaneously: first, downregulation in early survivor sepsis relative to healthy state; second, rebound from day 1 to day 5 among survivors; third, higher expression in survivors than in non-survivors at day 5; and fourth, absence of comparable recovery in non-survivors. Failure-related genes were required to satisfy two criteria simultaneously: first, higher expression in non-survivors than in survivors at day 5; and second, continued increase from day 1 to day 5 among non-survivors. Day 5 was used as the late disease-course anchor for gene selection because it provided the common endpoint of the discovery cohort; intermediate day 2–day 4 measurements were subsequently incorporated in trajectory modeling rather than ignored.

To improve interpretability of the gene set and reduce interference from extreme shifts in blood-cell composition, erythroid contamination-related genes were excluded before axis selection (19). Recovery-related genes were screened using the following logFC thresholds: logFC less than −0.2 for survivor day 1 relative to healthy controls, survivor time-course logFC greater than 0.2, day 5 survivor-versus-non-survivor logFC greater than 0.15, and non-survivor time-course logFC less than 0.15. Failure-related genes were screened using the following logFC thresholds: day 5 survivor-versus-non-survivor logFC less than −0.5 with adjusted significance, together with non-survivor time-course logFC greater than 0.2 and statistical support. These thresholds were chosen to prioritize directional consistency and moderate biological effect size across multiple contrasts rather than to optimize classification performance. The recovery thresholds were intentionally permissive because four independent direction rules had to be satisfied simultaneously, whereas the failure day 5 separation threshold and adjusted-significance requirement were stricter to reduce non-specific late injury signals. Candidate genes were then ordered using an explicit composite ranking. For recovery genes, the ranking score was calculated as (−min[early-sepsis logFC, 0]) + max[survivor time-course logFC, 0] + max[day 5 survivor–non-survivor logFC, 0] − max[non-survivor time-course logFC, 0]. For failure genes, the ranking score was (−day 5 survivor–non-survivor logFC) + max[non-survivor time-course logFC, 0] − max[survivor time-course logFC, 0]. The top 20 genes from each direction were retained to create a balanced 20+20 axis. Sensitivity analyses across 10–30 genes per direction were used to evaluate whether conclusions depended on this empirical threshold choice.

Within each cohort, axis-related genes were first standardized gene-wise, after which the recovery-failure score for each sample was calculated as the mean standardized expression of recovery-related genes minus that of failure-related genes. Because standardization was performed within each cohort, the present score is best interpreted as a relative coordinate for within-cohort position, temporal trajectory, and stratification differences rather than as a risk score with a shared absolute scale across cohorts. This design limits immediate single-patient deployment. A clinically deployable version would require a locked reference distribution from a training cohort, external calibration samples, or a rank-based single-sample implementation such as ssGSEA-like scoring, followed by prospective evaluation of fixed cutoffs.

### 2.4 External transfer validation of the recovery-failure axis

In GSE57065 and GSE65682, the same axis genes defined in the discovery cohort were used directly to calculate recovery-failure scores without retraining or feature replacement. In GSE57065, analyses focused on differences between healthy controls and patients with septic shock and on dynamic changes at 0, 24, and 48 h in lower- and higher-severity septic shock. In GSE65682, analyses focused on the relationships of the score with healthy state, 28-day outcome, and MARS molecular endotypes to determine whether the axis behaves more like a mortality label or a coordinate of host-state heterogeneity.

### 2.5 Analysis of associations with clinical severity scores

In bulk cohorts with publicly available severity annotations, we further assessed the relationship between the recovery-failure score and conventional bedside severity scores. GSE54514 provided baseline APACHE II information; therefore, Spearman correlation between the recovery-failure score and APACHE II was assessed in day 1 sepsis samples, and, among patients with paired day 1 and day 5 samples, changes in score from day 5 minus day 1 were correlated with baseline APACHE II and compared after median stratification. GSE57065 did not provide continuous SAPS II values but only high/low groupings; therefore, differences in recovery-failure scores between SAPS II strata were compared at 0, 24, and 48 h, as well as at the patient-level mean and last available score. Because bedside severity annotations were incomplete in public cohorts, these analyses were used only to assess complementarity between the axis and traditional severity scores rather than to define the axis itself.

### 2.6 Sensitivity analysis of axis robustness

To assess whether the recovery-failure axis depended on a specific gene-count threshold, we separately selected the top 10, 15, 20, 25, and 30 recovery-related candidate genes and failure-related candidate genes to construct alternative scores and repeated core comparisons in GSE54514, GSE57065, and GSE65682. Spearman correlations between each alternative score and the standard 20+20 score were then calculated to test whether the standard axis represented a stable host-state signal rather than a chance result under one particular threshold setting.

To test whether transferred score heterogeneity was larger than expected from arbitrary gene averaging, we performed a random-gene null analysis in GSE57065 and GSE65682. In GSE57065, serial samples were first standardized gene-wise within cohort and then averaged at the patient level; GSE65682 was analyzed at the patient-sample level because each patient contributed one sample. For each external cohort, 5,000 random gene scores were generated from genes common to both external platforms after excluding recovery-failure axis genes and erythroid-blacklist genes. Each random score matched the number of recovery and failure genes that actually mapped to that platform, namely 15 recovery and 16 failure genes in GSE57065 and 13 recovery and 16 failure genes in GSE65682. The observed patient-level score variance was compared with this null distribution using an empirical one-sided *P*-value.

To determine whether high-score patients shared broader transcriptomic structure beyond the score genes themselves, patients in GSE57065 and GSE65682 were divided into top and bottom tertiles according to the recovery-failure score. For all common non-axis and non-blacklisted genes, we calculated the high-score minus low-score expression effect within each cohort using standardized expression. Cross-cohort consistency was quantified by Spearman correlation of these non-axis gene effects and by 5,000 permutations that preserved high- and low-tertile group sizes. Overlap of the top 500 high-score-associated and low-score-associated non-axis genes was evaluated against hypergeometric expectation.

### 2.7 Single-cell data processing, integration, and cell annotation

Single-cell data from GSE167363 and GSE151263 were processed using the Seurat workflow (20). Quality-control criteria for GSE167363 were at least 200 detected genes and at most 6,000 detected genes per cell, total transcript counts no greater than 25,000, and mitochondrial transcript proportion no greater than 20%. For GSE151263, criteria were 200–7,000 genes per cell, total transcript counts no greater than 30,000, mitochondrial transcript proportion no greater than 20%, and hemoglobin transcript proportion no greater than 10%. After filtering, data were normalized using log-normalization, highly variable genes were identified, expression values were scaled, and principal component analysis was performed. Harmony was then used for batch correction and integration across samples or donors before shared-nearest-neighbor clustering and UMAP visualization. Major cell-state annotation was based on canonical marker expression and was reviewed in the integrated low-dimensional space. The same processing logic was applied to the monocyte-restricted object before substate scoring and visualization.

Cell annotation first relied on broad canonical marker-gene sets, including classical monocytes, antigen-presentation-related myeloid cells, interferon-activated myeloid cells, T cells, natural killer cells, platelets, and erythroid-related cells. These broad labels were used as an initial anchoring layer rather than as final high-resolution cell-type definitions. The recovery-failure axis was then projected onto the single-cell level, and score distributions, sample-level means, and temporal changes across cell states were compared to identify the principal carrier of the state signal. Classical monocytes were subsequently refined in a dedicated substate analysis.

### 2.8 Pseudobulk differential analysis and pathway enrichment

In GSE167363, pseudobulk samples were constructed by donor, time point, and cell state, requiring at least 20 cells per donor-state combination. This design used the donor as the statistical unit to reduce pseudoreplication bias inherent to single-cell analysis. After low-expression genes were filtered with edgeR (21) and data were transformed with voom (22), differential analysis was performed using limma linear modeling, with emphasis on state differences between non-survivors and survivors at 6 h after onset. On a donor-paired basis, temporal changes were also compared separately within survivors and non-survivors. At the pathway level, MSigDB Hallmark gene sets (23) were analyzed with preranked fgsea (24), and normalized enrichment scores were used to describe program upregulation or downregulation.

### 2.9 Substate refinement of classical monocytes

Within GSE167363, classical monocytes were further subjected to reintegration and reclustering. Substate labels were not defined as discrete UMAP clusters; instead, they represented dominant functional programs assigned on the basis of three prespecified gene sets: proinflammatory, interferon-activated, and antigen-presentation programs (25, 26). Each monocyte was assigned a functional-state label according to its highest program score, and sample-level differences in substate composition and recovery-failure scores were compared to evaluate the association between each program-defined substate and displacement along the state axis.

### 2.10 Monocyte-related ligand-receptor communication analysis

Based on public ligand-receptor resources, ligand-receptor pairs supported by multiple mainstream databases were selected (27), and communication from monocyte substates to T cells and platelets was specifically evaluated in GSE167363. Interactions were scored only when both sending and receiving states contained at least 10 cells in a sample and when more than 10% of cells expressed both the ligand and the receptor. Communication strength was defined as the log-transformed product of mean expression in the sending and receiving populations. Differences between survivors and non-survivors at 6 h and within-donor changes from 0 to 6 h were then compared.

### 2.11 Longitudinal mixed-effects modeling and adjustment for cell composition

To make fuller use of all repeated measurements from day 1 to day 5 in GSE54514, we fitted linear mixed-effects models (28) with patient_id as a random intercept and day as a continuous time variable to test the interaction between time and outcome. To assess the influence of whole-blood cell-composition shifts, an adjustment model based on neutrophil proportion from the public metadata was constructed; among samples with complete annotations, baseline APACHE II was further included as a covariate. Robustness of trajectory divergence in the discovery cohort was evaluated by comparing models with and without the interaction term and by reporting fixed-effect estimates and confidence intervals.

### 2.12 Single-cell leave-one-donor-out robustness assessment

Given the limited number of donors in GSE167363, leave-one-donor-out analyses (29) were performed at the 6-h time point for major immune states, original Seurat clusters, and classical monocyte substates. For major immune states and original clusters, donor-level mean absolute axis values and their rankings were calculated. The original-cluster screen was added to address whether broad T-cell annotation might mask a T-cell subcluster with a stronger recovery-failure pattern. Cluster-level sample means were interpreted only when a donor-sample cluster contained at least 20 cells. For outcome-shift comparisons, a cluster was considered well-covered only when at least two survivor donors and two non-survivor donors were represented, while all clusters were retained in Supplementary material for transparency. For monocyte substates, sample-level mean axis differences between non-survivors and survivors were compared, and the direction, ranking, and estimability of the effect after removing each donor in turn were recorded to assess whether the conclusions depended on any single donor.

### 2.13 Statistical analysis and reproducibility

Between-group comparisons were performed using the Wilcoxon rank-sum test, limma linear models, paired-design models, random-gene permutation tests, or rank-correlation analyses as appropriate for the analysis target. The Benjamini-Hochberg method was used to control the false discovery rate in multiple-comparison settings. All data cleaning, statistical analyses were performed in R and Python.

## 3 Results

### 3.1 The overall study design enabled layered validation from dynamic axis construction to single-cell anchoring

**FIGURE 1:**
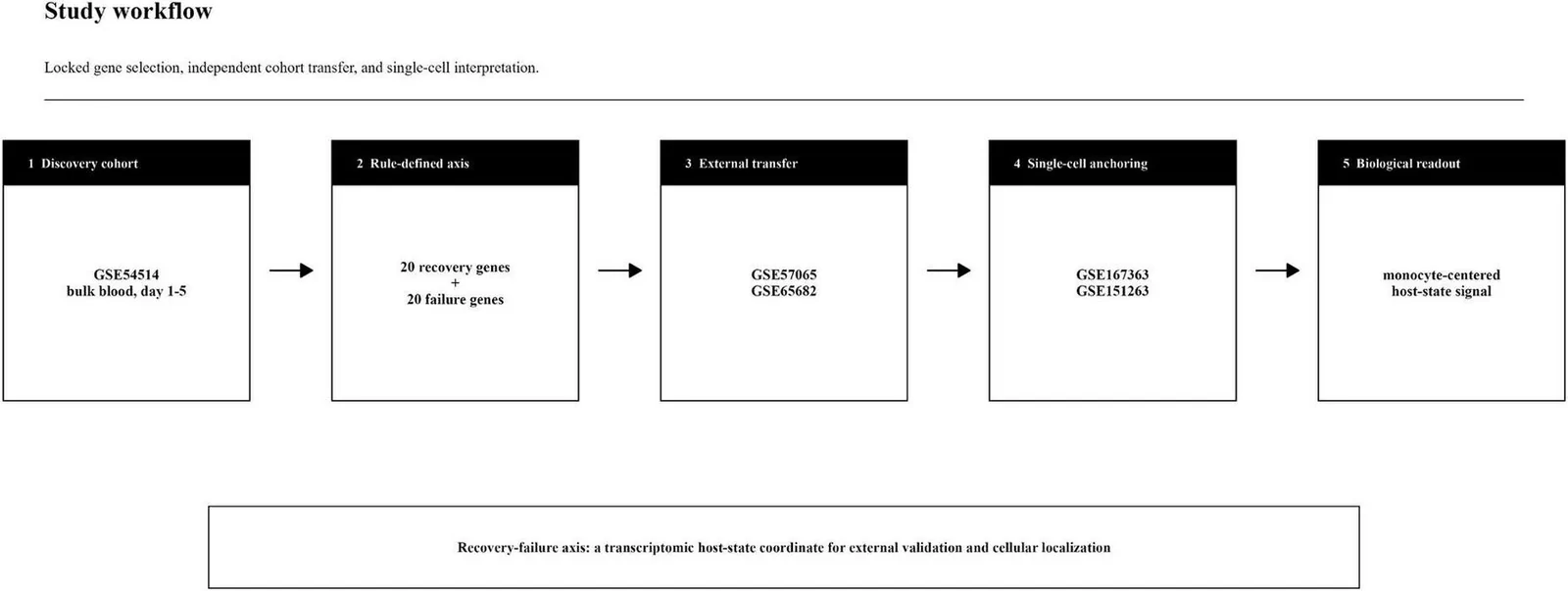
Study architecture and conceptual framework of the sepsis recovery-failure project. The simplified workflow summarizes the analytical sequence from GSE54514 whole-blood discovery to rule-defined 20+20 recovery-failure axis construction, external bulk transfer in GSE57065 and GSE65682, single-cell anchoring in GSE167363 and GSE151263, and biological readout centered on monocyte-related host-state signals.

We adopted a five-layer analytical framework of “discovery-transfer-anchoring-refinement-generalization.” We first defined the recovery-failure axis in GSE54514 using longitudinal whole-blood data; next, we validated its cross-cohort transferability and its ability to stratify host-state heterogeneity in GSE57065 and GSE65682; we then identified its principal single-cell carrier and characterized associated programs in GSE167363; classical monocytes were further refined into distinct functional states and combined with cell-communication analysis; finally, GSE151263 was used to examine supplementary heterogeneity information for an external adverse endpoint (Figure 1).

### 3.2 The recovery-failure axis was jointly defined by multidimensional dynamic evidence

**FIGURE 2:**
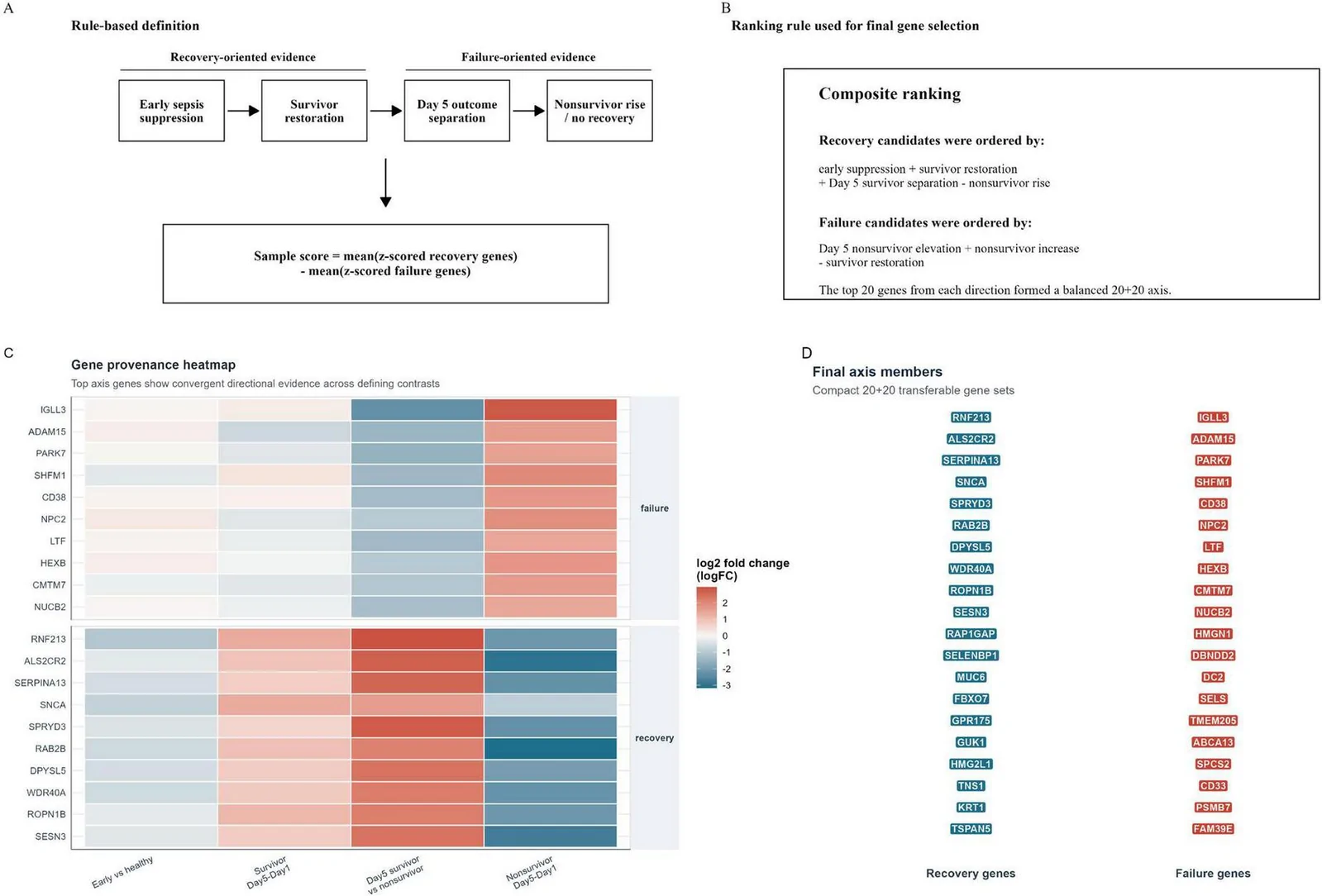
Transparent construction of the sepsis recovery-failure axis. **(A)** Rule-based definition of recovery and failure evidence. Recovery genes required early suppression, survivor restoration, day 5 survivor and non-survivor separation, and absence of equivalent non-survivor recovery. Failure genes required day 5 non-survivor elevation and non-survivor increase over time. **(B)** Composite ranking used for final gene selection. **(C)** Gene provenance heatmap showing directional evidence across the defining contrasts. **(D)** Final 20 recovery and 20 failure genes used for the transferable score. The final sample score was defined as mean (z-scored recovery genes) minus mean (z-scored failure genes).

In the discovery cohort GSE54514, the recovery-failure axis was not obtained from a single direct comparison between survivors and non-survivors. Instead, it was constrained by four categories of recovery evidence and two categories of failure evidence, including early sepsis deviation, time-dependent recovery, late disease-course outcome separation, and absence of recovery in non-survivors. Using the prespecified thresholds and erythroid-gene exclusion strategy, we ultimately obtained 20 recovery-related genes and 20 failure-related genes (Figure 2).

The key value of this construction strategy is that it encodes “disease-course direction” into the score definition itself. In other words, selected genes must not only distinguish survivors from non-survivors but also display opposite recovery-versus-failure trajectories over time. Thus, from the moment it is defined, the recovery-failure axis functions as a dynamic state coordinate rather than a static risk score.

### 3.3 Survivors and non-survivors showed opposite disease-course trajectories in the discovery cohort

**FIGURE 3:**
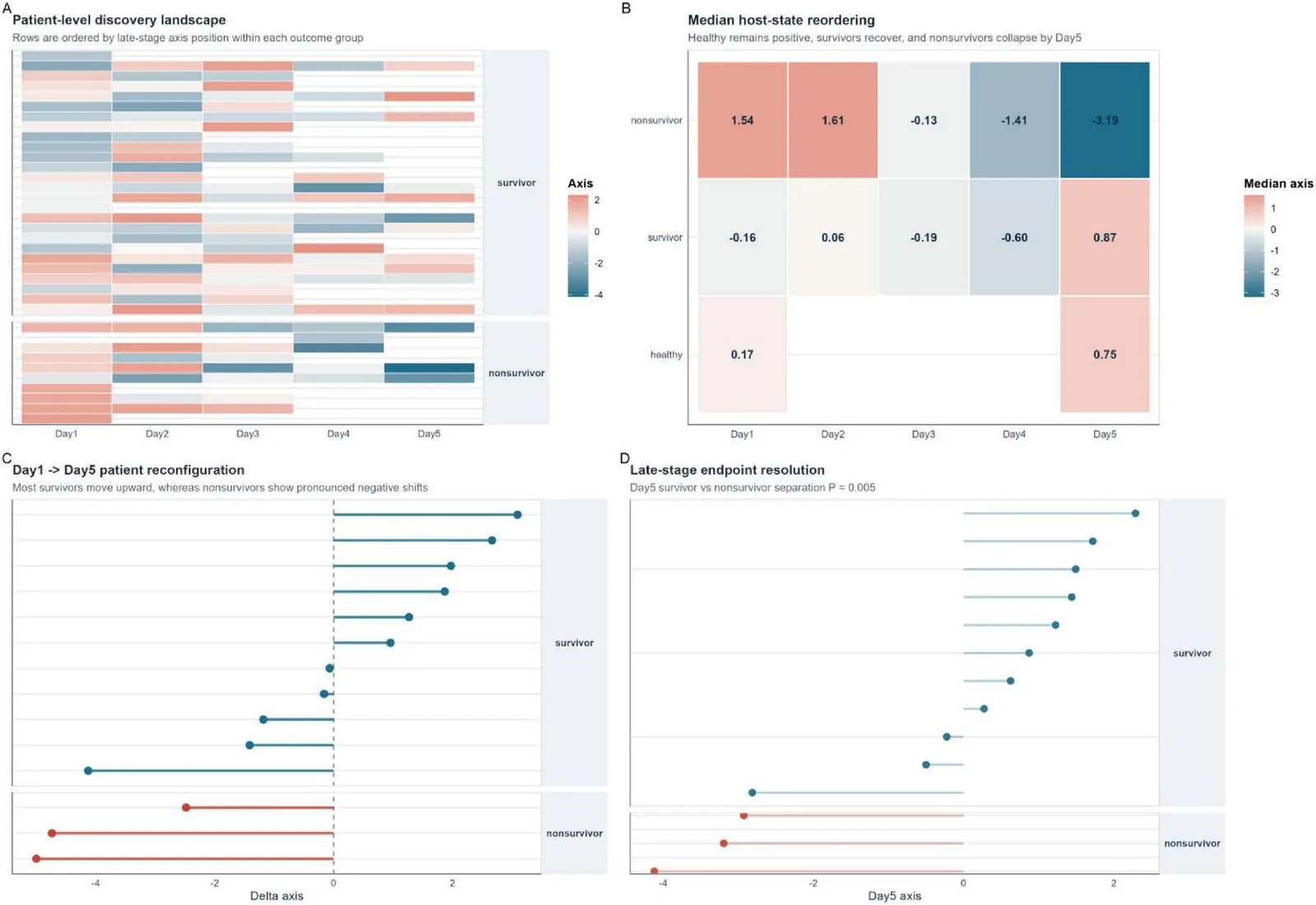
Bulk discovery reveals opposite host-state trajectories in survivors and non-survivors. **(A)** Patient-level heatmap ordered by late-stage axis position within each outcome group. **(B)** Median axis values from day 1 to day 5. Survivors showed a transient mid-course decrease but rebounded by day 5, whereas non-survivors shifted progressively toward the failure end after day 2. **(C)** Individual day 5 minus day 1 changes. **(D)** Day 5 endpoint separation between survivors and non-survivors (*P* = 0.005).

In GSE54514, survivors and non-survivors showed different disease-course movements, but the survivor trajectory was not strictly monotonic. Survivor median recovery-failure scores were −0.163 on day 1, 0.064 on day 2, −0.190 on day 3, −0.600 on day 4, and 0.874 on day 5. Thus, survivors showed a transient mid-course decrease followed by a late rebound above the day 1 level. By contrast, non-survivor median scores were 1.542 on day 1, 1.610 on day 2, −0.134 on day 3, −1.414 on day 4, and −3.195 on day 5, indicating progressive displacement toward the failure end of the axis after day 2. The day 5 versus day 1 comparison was statistically significant in non-survivors and showed the expected positive direction in survivors; by day 5, the two groups were clearly separated (Figure 3).

The mid-course decline among survivors likely reflects early host-state instability rather than true failure of recovery. One possible interpretation is that sepsis survivors pass through a transient immunometabolic contraction or treatment-response phase before recovery-related programs become consolidated by day 5. Therefore, the axis should not be interpreted as a monotonic daily biomarker. Its more appropriate interpretation is trajectory direction and late-course resolution: non-survivors failed to convert early activation into a durable recovery program, whereas survivors ultimately reoriented toward the recovery end of the state axis.

When all repeated measurements from day 1 to day 5 were further incorporated into a linear mixed-effects model, a significant interaction between time and outcome was observed (beta = −1.164, 95% CI −1.561 to −0.768, *P* < 0.001). After adjustment for neutrophil proportion from the public metadata, the interaction remained stable (beta = −1.131, 95% CI −1.536 to −0.726, *P* < 0.001). Results were essentially unchanged after further adjustment for baseline APACHE II (beta = −1.133, 95% CI −1.539 to −0.726, *P* < 0.001), whereas neither neutrophil proportion nor APACHE II itself reached statistical significance. These findings indicate that trajectory divergence in the discovery cohort cannot be adequately explained by simple shifts in neutrophil proportion or by baseline severity differences and provide a complementary full-time-course assessment beyond the day 5 versus day 1 gene-selection anchor (Supplementary Figure 3).

### 3.4 The recovery-failure axis transferred to independent whole-blood cohorts but more closely resembled a coordinate of host-state heterogeneity

**FIGURE 4:**
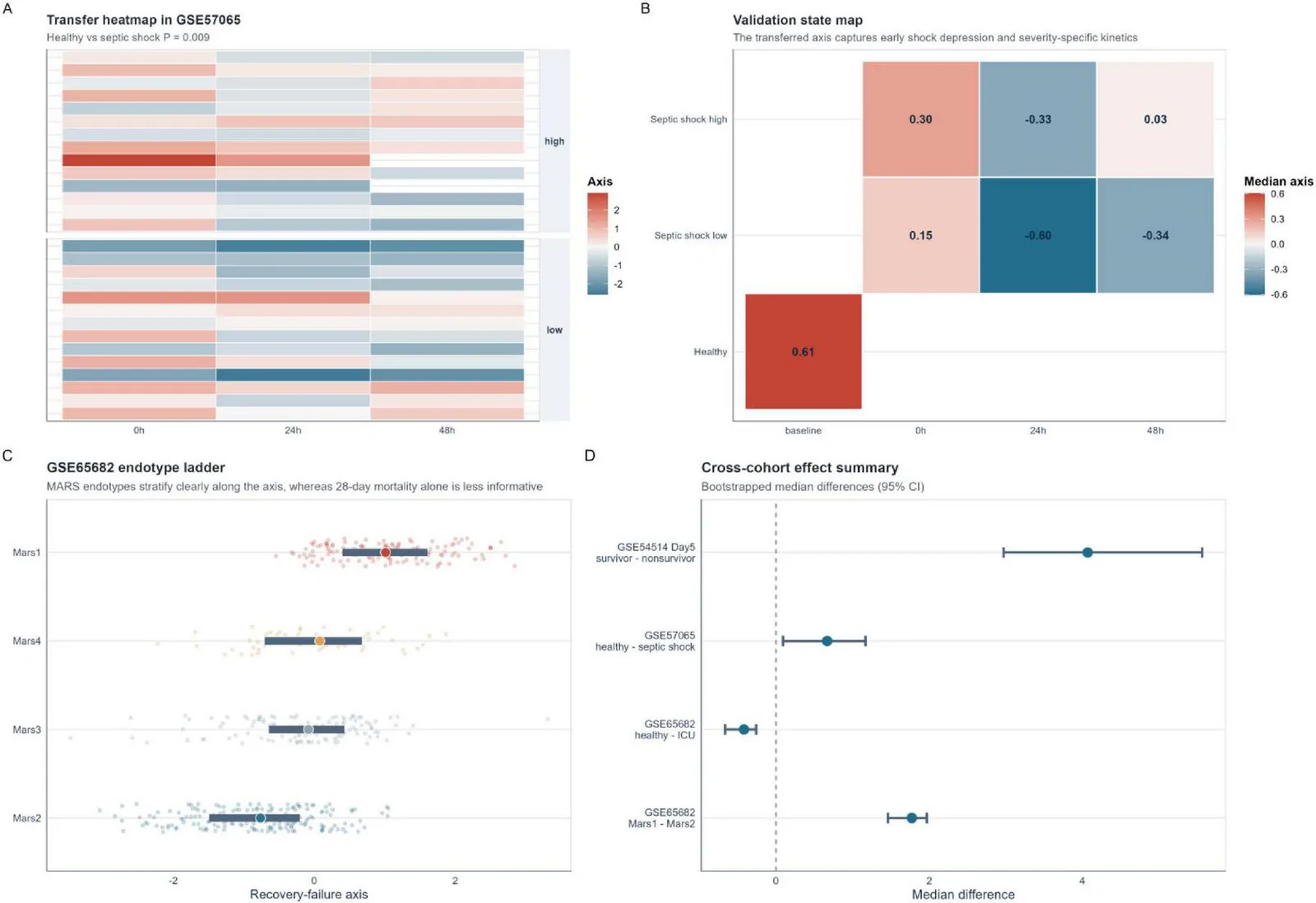
External bulk validation supports transferability and host-state heterogeneity of the axis. **(A)** Transfer heatmap in GSE57065. Patients with septic shock were separated from healthy controls (*P* = 0.009). **(B)** Validation state map. Low and high denote the public SAPS II severity strata; high-versus-low differences were descriptive and were not statistically significant at the tested time points. **(C)** GSE65682 Molecular Diagnosis and Risk Stratification (MARS) endotype ladder, showing stronger molecular endotype stratification than 28-day mortality alone. **(D)** Cross-cohort effect summary with bootstrapped median differences and 95% confidence intervals. The GSE65682 healthy-intensive care unit (ICU) direction should be interpreted cautiously because the score was standardized within cohort rather than calibrated to a universal absolute scale.

In GSE57065, 82 serial samples from 28 patients with septic shock were separated from 25 healthy controls. The median recovery-failure score was 0.607 in healthy controls and −0.061 in the septic shock group (Wilcoxon *P* = 0.009), indicating that the transferred axis retained a disease-state signal in an independent clinical setting. In this cohort, “low” and “high” refer to the public SAPS II severity strata rather than newly defined molecular groups. The high-SAPS II group had a higher median score at 0 h than the low-SAPS II group (0.30 versus 0.15), followed by a decrease at 24 h and partial rebound at 48 h; however, high-versus-low SAPS II differences were not statistically significant at any measured time point. We therefore interpret these severity-specific kinetics descriptively rather than as a validated severity-group separation (Figure 4).

In GSE65682, 760 intensive care unit patients were significantly separated from 42 healthy controls. The direction of the healthy-versus-ICU difference in this cohort differed from GSE57065 when scores were standardized within cohort, emphasizing that the current implementation should not be read as a universal absolute scale. Notably, the recovery-failure axis did not significantly distinguish 28-day death in GSE65682 (*P* = 0.214). However, among the 479 samples with complete endotype information, the axis formed a clear gradient across MARS classes, with the highest median value in Mars1, the lowest in Mars2, and intermediate values in Mars3 and Mars4. This pattern suggests that the recovery-failure axis is intrinsically closer to a coordinate of host-state heterogeneity than to a simple surrogate label for death.

Accordingly, cross-cohort performance in this study should be understood as transfer of a biologically interpretable stratifying signal rather than as preservation of a fixed absolute cutoff or a single mortality-prediction direction across all cohorts. In a syndrome as heterogeneous as sepsis, a host-state coordinate may be more biologically informative than a score forced to maximize mortality prediction across datasets with different platforms, sampling designs, and case mixtures.

### 3.5 The recovery-failure axis showed only limited correlation with public clinical severity scores, suggesting complementary information beyond bedside scoring

In GSE54514, which included baseline APACHE II annotations, the recovery-failure score in day 1 sepsis samples showed only a limited positive correlation with APACHE II, with a Spearman correlation coefficient of 0.279 (*P* = 0.104). After median stratification by APACHE II, the high-score group had a higher median day 1 recovery-failure score, but the difference was not statistically significant, with a median difference of 0.857 (*P* = 0.110). Among patients with paired day 1 and day 5 samples, baseline APACHE II showed a negative trend with the day 5 minus day 1 change in recovery-failure score (Spearman correlation coefficient, −0.271; *P* = 0.349). The high-APACHE II group also showed a smaller recovery increment, but the difference again did not reach significance, with a median difference of −2.680 (*P* = 0.147).

In GSE57065, which included only high/low SAPS II group annotations, no significant separation was observed between SAPS II strata at 0, 24, or 48 h, nor at the patient-level mean or last available score; all *P*-values were greater than 0.32. These findings indicate that the recovery-failure axis does not simply recapitulate traditional bedside severity scores but more likely captures host transcriptomic-state information complementary to APACHE II or SAPS II. The practical value of such complementarity would be to stratify molecular host states within clinically similar patients, monitor direction of change during early treatment, and nominate state-linked biological programs for trial enrichment or mechanistic testing, rather than to replace existing clinical scores (Supplementary Figure 1).

### 3.6 Sensitivity analysis of gene-set size supported the robustness of the recovery-failure axis definition

When the numbers of included recovery-related and failure-related genes were varied to 10, 15, 20, 25, and 30, the same directional pattern was preserved in GSE54514: scores increased in survivors, decreased in non-survivors, and the two groups remained significantly separated on day 5. Across these settings, the day 5 minus day 1 difference in survivors ranged from 0.952 to 1.146, with *P*-values from 0.040 to 0.060; in non-survivors, the day 5 minus day 1 difference ranged from −4.929 to −4.501, with *P* = 0.009 under all settings; and the day 5 difference between survivors and non-survivors ranged from 3.905 to 4.162, with *P* = 0.005 under all settings.

In external cohorts, the difference between healthy controls and septic shock in GSE57065 ranged from 0.567 to 0.820, with *P*-values from <0.001 to 0.015, while the difference between healthy controls and ICU patients in GSE65682 ranged from −0.751 to −0.256, with *P*-values from <0.001 to 0.039. By contrast, the comparison between 28-day survivors and non-survivors in GSE65682 remained unstable across settings, with *P*-values ranging from 0.117 to 0.883. Meanwhile, Spearman correlations between each alternative score and the standard 20+20 score were high across the three bulk cohorts, reaching 0.989–0.998, 0.938–0.992, and 0.949–0.991, respectively. Supplementary Figure 2 presents effect-size and rank-correlation sensitivity analyses across gene-set sizes; it is not intended to reproduce the day-by-day trajectory plot in Figure 3. These results indicate that the standard recovery-failure axis captures a stable host-state signal rather than representing an accidental construct driven by a specific gene-count threshold (Supplementary Figure 2).

We next tested whether the observed external-cohort heterogeneity exceeded what would be expected if arbitrary genes were averaged into a similar score. In GSE57065, the patient-level variance of the observed recovery-failure score was 0.889, compared with a matched random-gene null mean of 0.073 and a 95th percentile of 0.135; the observed variance was 12.2-fold higher than the null mean (empirical *P* = 0.0002). In GSE65682, the observed variance was 1.172, compared with a null mean of 0.132 and a 95th percentile of 0.220; the observed variance was 8.9-fold higher than the null mean (empirical *P* = 0.0002). These results indicate that the transferred score did not behave like a typical random average that cancels out in external cohorts (Supplementary Figure 5).

We also asked whether patients with high scores in different external cohorts shared transcriptomic structure beyond the genes used to calculate the score. After excluding all recovery-failure axis genes and erythroid-blacklist genes, high-score versus low-score effects across 10,270 common non-axis genes were strongly concordant between GSE57065 and GSE65682 (Spearman rho = 0.718; permutation *P* = 0.0002). Among the top 500 high-score-associated non-axis genes, 202 overlapped between cohorts, compared with 24.3 expected by chance (hypergeometric *P* = 1.59 × 10^–144^). Among the top 500 low-score-associated non-axis genes, 172 overlapped, again far above the 24.3 expected by chance (hypergeometric *P* = 7.85 × 10^–^107). Thus, the score-defined heterogeneity reflected a reproducible broader host-transcriptomic structure, not merely different numerical scores across cohorts (Supplementary Figure 5).

### 3.7 Single-cell anchoring indicated that classical monocytes are the principal immune-state carrier of the recovery-failure axis

**FIGURE 5:**
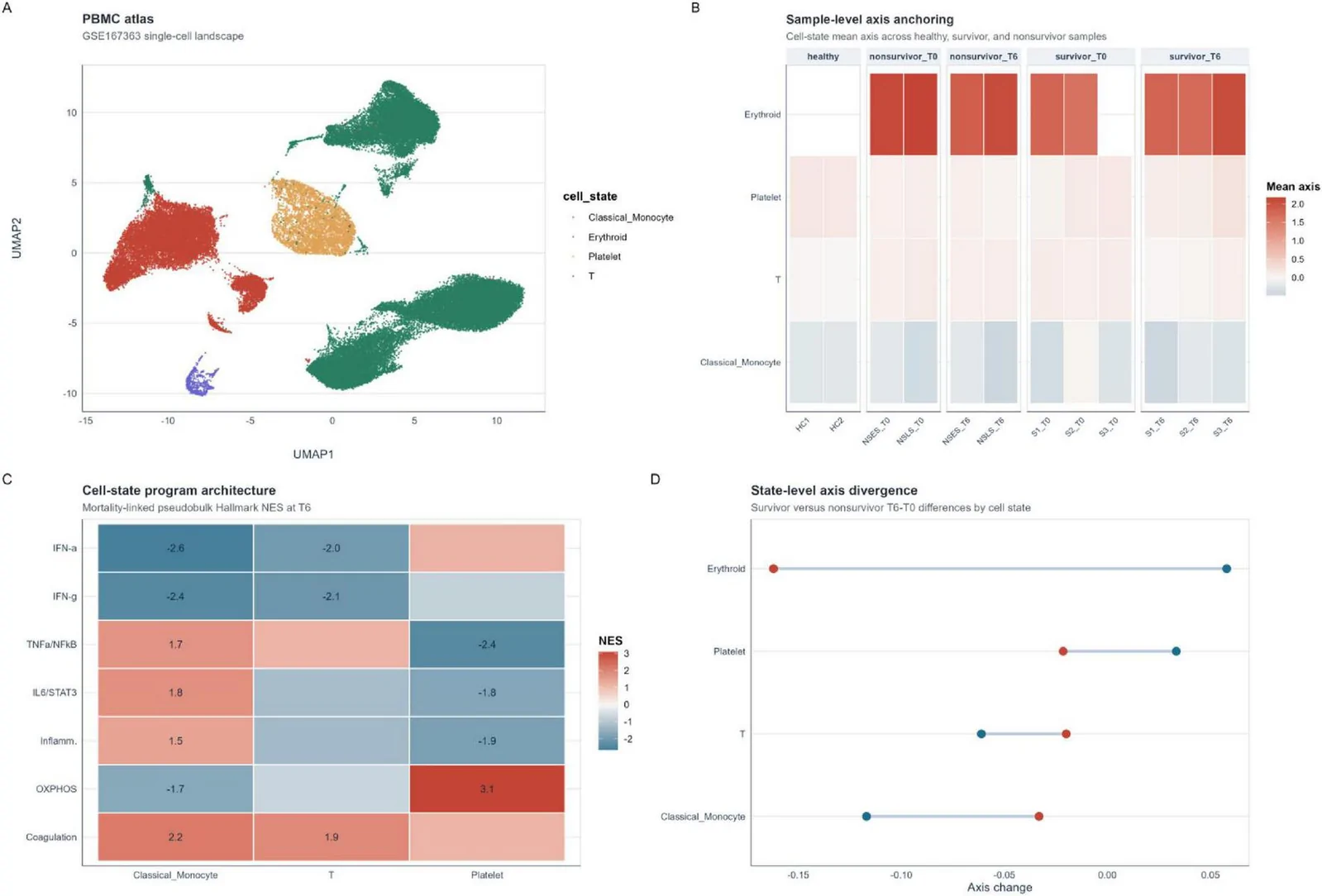
Single-cell anchoring localizes the strongest recovery-failure axis signal to classical monocytes. **(A)** Broad PBMC atlas from GSE167363. The annotation was intentionally coarse for initial anchoring and was followed by monocyte-focused refinement in Figure 6. **(B)** Sample-level axis anchoring across major cell states. Erythroid-related cells could show high projected values despite erythroid-gene exclusion, but this small compartment was not interpreted as the principal carrier. **(C)** Mortality-linked pseudobulk Hallmark enrichment at 6 h, showing reduced interferon (IFN)-alpha, IFN-gamma, and oxidative phosphorylation together with increased TNFA/NFkB, IL6/STAT3, inflammatory response, and coagulation programs in non-survivor classical monocytes. **(D)** State-level survivor versus non-survivor T6–T0 axis divergence by major cell state.

In GSE167363, 59,227 cells were retained after quality control, including 15,730 from healthy controls, 16,439 from non-survivors, and 27,058 from survivors. The initial PBMC map used broad major-state annotation and was therefore expected to contain heterogeneity within the T-cell and monocyte compartments. This broad layer was used only to ask where the bulk-defined axis signal was most prominent. After projection of the recovery-failure axis to the single-cell level, classical monocytes showed the largest donor-level absolute axis values among major immune states at 6 h (Figure 5).

Further leave-one-donor-out analysis showed that classical monocytes consistently ranked first in absolute axis value among major immune states regardless of which sepsis donor was removed, whereas T cells and platelets consistently ranked behind them (Supplementary Figure 4). Erythroid-related cells showed high positive mean scores in some samples despite exclusion of erythroid contamination genes from axis selection. This does not contradict the blacklist step: the blacklist prevented canonical erythroid transcripts from defining the axis, whereas cell-level projection can still produce high scores in a small erythroid-related compartment because of residual expression of non-blacklisted axis genes and sparse cell coverage. We therefore do not interpret erythroid-related cells as the main biological carrier of the axis. Given the limited number of single-cell donors, the evidence is better interpreted as cellular anchoring to classical monocytes rather than exclusive causal attribution.

Because broad T-cell annotation could theoretically mask opposing or concentrated T-cell subcluster signals, we re-examined the original Seurat clusters in GSE167363. At the T6 sepsis time point, broad classical monocytes had a mean absolute sample-level axis value of 0.338, whereas broad T cells had a mean absolute value of 0.080. In the original-cluster ranking, erythroid cluster g8 ranked first but remained a small compartment not used for axis interpretation; the next three positions were all classical monocyte clusters (g11, g1, and g5). Nine T-cell clusters were tested directly. The highest-ranking T-cell cluster by absolute axis value was T_g12, which ranked fifth overall but was represented only in survivor donors at T6. A small T_g14 cluster showed a nominal survivor and non-survivor difference, but it was supported by only one survivor donor and one non-survivor donor. Under the prespecified coverage rule requiring at least two survivor and two non-survivor donors, the strongest T-cell cluster shift was T_g0 and remained smaller than the strongest monocyte-cluster shift. In leave-one-donor-out ranking, classical monocyte clusters g11 and g1 remained in the top three in 4 of 5 donor-removal iterations, whereas no T-cell cluster did so more than once. These analyses do not exclude a role for T cells in sepsis biology, but they did not support redirecting the downstream axis-anchoring analysis from monocytes to T-cell subclusters (Supplementary Figure 6).

### 3.8 Classical monocytes from non-survivors displayed an associated program of “insufficient interferon support, persistent inflammation, and metabolic impairment”

In pseudobulk analysis of GSE167363, the comparison of classical monocytes between survivors and non-survivors at 6 h after onset most clearly revealed programs associated with adverse outcome. In classical monocytes from non-survivors, interferon-alpha and interferon-gamma responses were significantly reduced, with normalized enrichment scores of −2.647 and −2.433, respectively. Tumor necrosis factor/NF-kB and IL6-JAK-STAT pathways increased to 1.742 and 1.816, accompanied by coordinated enhancement of inflammatory and coagulation programs, while oxidative phosphorylation was significantly downregulated, with a normalized enrichment score of −1.705 (Figure 5).

These findings suggest that poor outcome cannot be adequately summarized by the overly coarse statement that “inflammation is higher.” More precisely, classical monocytes in non-survivors were linked to an unstable state characterized by insufficient protective interferon support, persistent dominance of inflammatory and injury programs, and impaired energy metabolism. That is, the problem is not merely amplification of inflammatory signaling, but also failure to establish recovery-promoting and convergent programs in time.

### 3.9 Functional program branches existed within classical monocytes, with the interferon-activated subpopulation warranting particular attention

**FIGURE 6:**
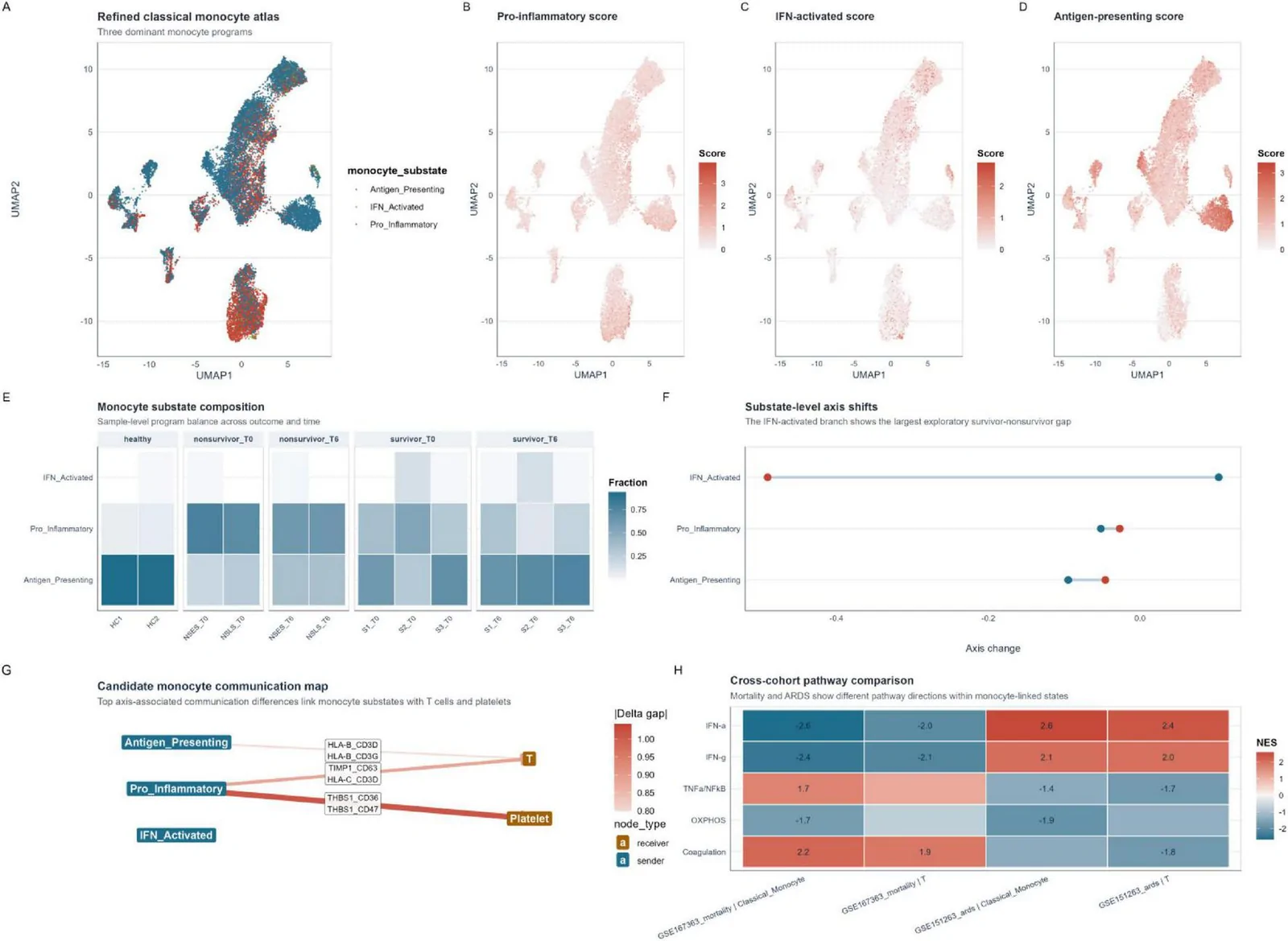
Monocyte-state refinement and candidate communication analyses provide exploratory context for the axis signal. **(A)** Refined classical monocyte atlas. Program labels represent dominant functional scores rather than discrete UMAP clusters. **(B–D)** Pro-inflammatory, IFN-activated, and antigen-presenting program scores. **(E)** Sample-level monocyte substate composition across outcome and time. **(F)** Substate-level survivor-non-survivor axis shifts; the interferon (IFN)-activated branch showed the largest exploratory gap but had limited cellular coverage. **(G)** Candidate monocyte communication map linking monocyte substates with T cells and platelets. **(H)** Cross-cohort pathway comparison showing that the external acute respiratory distress syndrome (ARDS) cohort did not reproduce the mortality-associated pathway direction.

Further program-based subdivision of classical monocytes identified three dominant functional labels: antigen-presentation, proinflammatory, and interferon-activated, comprising 10,988, 5,443, and 321 cells, respectively. These substates should be interpreted as program-score assignments over a continuous monocyte landscape, not as discrete UMAP clusters. Although the interferon-activated subpopulation was the smallest, it showed the most pronounced non-survivor-versus-survivor axis displacement in the full dataset. Additional leave-one-donor-out analysis showed that in 4 of the 5 estimable iterations, the interferon-activated substate exhibited the largest displacement magnitude. When the non-survivor donor NSES was excluded, the comparison became non-estimable because the remaining non-survivor at 6 h no longer retained this substate (Figure 6 and Supplementary Figure 4).

These findings suggest that recovery capacity in sepsis may not depend solely on changes in total monocyte abundance or on the degree of proinflammation alone, but may instead relate to whether a small emergency interferon-associated program can be mobilized and maintained. However, the IFN-activated substate contained only 321 cells in the analyzed GSE167363 object and became non-estimable in one leave-one-donor-out iteration after removal of the NSES donor. Therefore, this branch should be interpreted as a hypothesis-generating signal requiring validation in larger mortality-linked single-cell cohorts, not as a definitive mechanistic mediator.

### 3.10 Exploratory candidate interaction differences were observed between monocytes and T cells or platelets

In ligand-receptor communication analysis, we focused on signaling output from monocyte substates to T cells and platelets. Multiple recovery-associated communication axes were preserved in survivors but were clearly attenuated in non-survivors. Among them, the THBS1-CD36 axis from proinflammatory monocytes to platelets showed the largest difference, followed by THBS1-CD47. The TIMP1-CD63 axis from proinflammatory monocytes to T cells, together with human leukocyte antigen complex- and beta2-microglobulin-related signals, also showed more marked decreases in non-survivors (Figure 6).

These findings suggest that axis-related differences are not limited to intracellular transcriptional programs within monocytes but may also involve altered ability of monocytes to transmit key information to the adaptive immune system and platelet compartment. Although limited by donor number, these results provide exploratory evidence and, importantly, nominate candidate interactions for subsequent experimental validation.

### 3.11 The external single-cell cohort provided supplementary heterogeneity information rather than concordant replication

**FIGURE 7:**
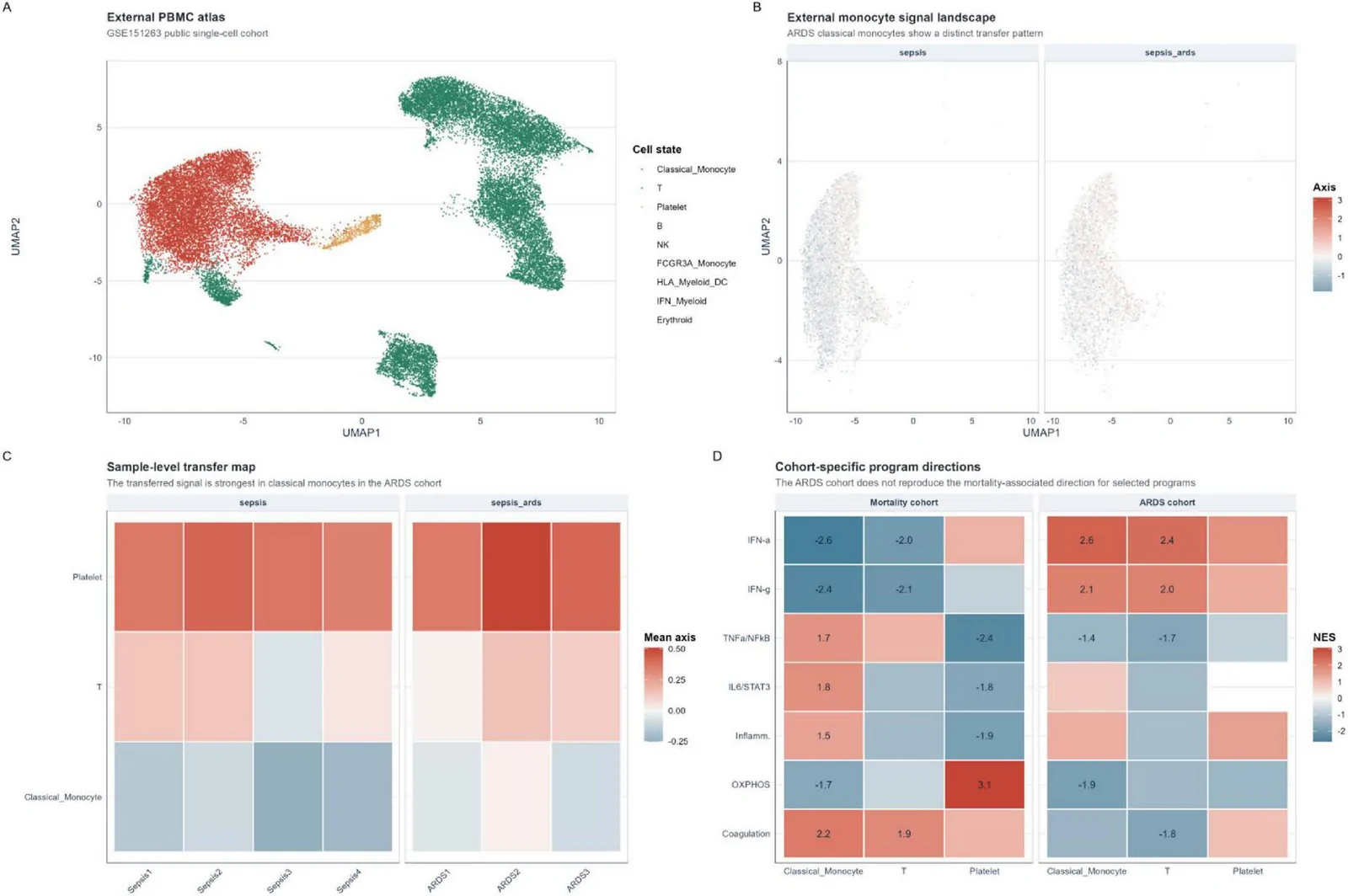
External acute respiratory distress syndrome (ARDS) single-cell data show classical-monocyte anchoring but do not reproduce the mortality-associated direction. **(A)** External PBMC atlas from GSE151263. **(B)** Classical-monocyte signal landscape in sepsis and sepsis with ARDS. **(C)** Sample-level transfer map showing that the transferred signal remained prominent in classical monocytes. **(D)** Cohort-specific program directions. The ARDS cohort supported endpoint heterogeneity rather than strict mortality-direction replication.

In GSE151263, 24,796 cells were retained after quality control, including 15,099 from uncomplicated sepsis and 9,697 from sepsis with acute respiratory distress syndrome. The dominant cell groups were again T cells and classical monocytes, with 14,830 and 9,455 cells, respectively. In this cohort as well, the recovery-failure axis showed its most prominent state signal in classical monocytes, suggesting that external adverse states and the mortality-related cohort may share monocyte-related features (Figure 7).

However, unlike the mortality-related adverse state in GSE167363, classical monocytes in GSE151263 more closely followed a program pattern of “increased interferon response with decreased oxidative phosphorylation,” and the difference in recovery-failure score showed only a marginal trend. This means that the cohort did not reproduce the direction observed in the mortality cohort and therefore should not be described as strict external validation. A more cautious interpretation is to treat it as supplementary evidence of endpoint heterogeneity, indicating that different adverse endpoints may correspond to different program trajectories.

**FIGURE 8:**
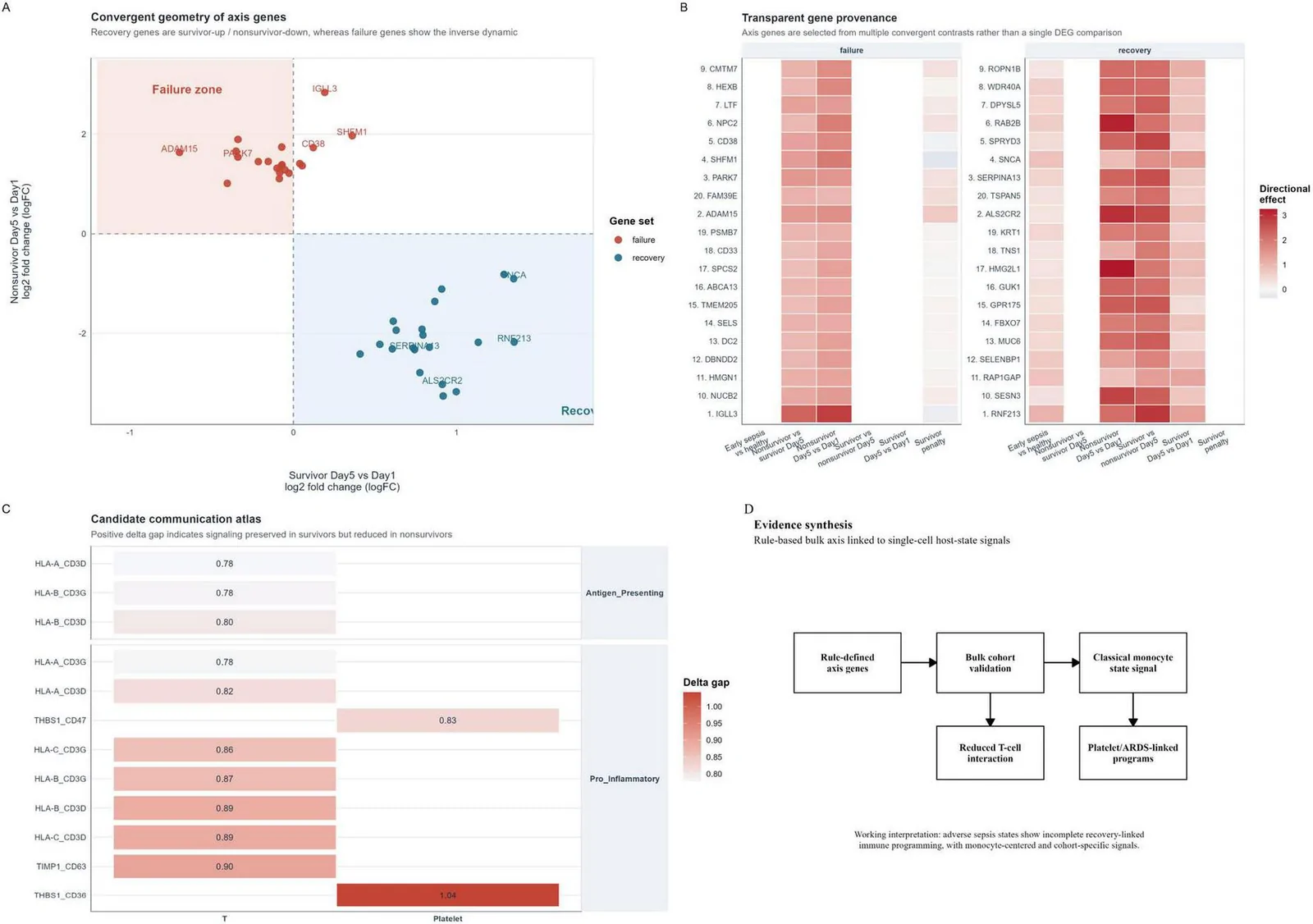
Transparent axis construction and multi-layer evidence support a rule-based recovery-failure host-state score. **(A)** Convergent geometry of recovery and failure genes in survivor and non-survivor longitudinal contrasts. **(B)** Transparent gene provenance across the defining contrasts. **(C)** Candidate communication atlas summarizing recovery-linked receptor-ligand axes from monocytes to T cells and platelets. **(D)** Evidence synthesis connecting the bulk-defined axis to a shared monocyte-centered recovery barrier and cohort-specific adverse-state branches.

This result expands the overall framework from a single-endpoint explanation to a more cautious view in which endpoint heterogeneity exists beyond the mortality-associated pattern, which is also more consistent with the true biology of sepsis as a complex syndrome (Figure 8).

## 4 Discussion

The core value of this study lies not in building yet another diagnostic or mortality prediction model for sepsis, but in proposing a host-state framework grounded in disease-course dynamics. Our contribution is more specific: the recovery-failure axis integrates early deviation, survivor recovery, late outcome separation, and absence of recovery in non-survivors, then connects that rule-based whole-blood state coordinate to external cohorts and single-cell cellular anchoring. In response to the concern that any gene set may appear heterogeneous across patients, we added random-gene null testing and non-axis transcriptomic concordance analyses. These analyses showed that the external-cohort variance of the transferred score was substantially greater than matched random-gene scores and that high-score patients in independent cohorts shared broad non-axis transcriptional structure. The axis is therefore better viewed as a process-oriented coordinate of a reproducible host-state structure than as a simple list of genes with variable expression.

First, this framework received support at the whole-blood level, with important qualifications. In the discovery cohort, survivors and non-survivors displayed divergent trajectories, and the mixed-effects model incorporating all day 1 to day 5 measurements confirmed a strong time-by-outcome interaction (30–32). The non-monotonic survivor pattern between day 2 and day 4 argues against treating the score as a simple daily monotonic biomarker; instead, the axis is most informative as a trajectory and late-course resolution coordinate. After adjustment for neutrophil proportion and baseline APACHE II, the interaction remained stable, suggesting that the trajectory split cannot be adequately explained by simple cell-composition shifts or baseline severity differences.

Analysis of associations with clinical severity scores further helped define the translational positioning of the axis. In currently available cohorts with public APACHE II or SAPS II annotations, the recovery-failure axis showed only limited association with conventional bedside severity scores. This finding does not by itself prove clinical utility, but it supports non-redundancy. A realistic future use would be to combine the axis with clinical scores to identify molecularly different host states among patients with similar bedside severity, monitor whether a patient is moving toward or away from recovery, enrich trials for patients with a specific host-state abnormality, and generate hypotheses about monocyte interferon, inflammatory, metabolic, or coagulation programs (33, 34).

Second, single-cell analyses provided a cellular anchor centered on classical monocytes for this state axis. The initial PBMC annotation was deliberately broad, and Figure 6 provides the higher-resolution monocyte refinement. We also added an original-cluster-level screen to address whether a coarse T-cell label could hide a stronger T-cell subcluster signal. Although individual sparse T-cell clusters showed nominal differences, the absolute axis ranking and leave-one-donor-out stability favored classical monocyte clusters, whereas well-covered T-cell clusters did not exceed the strongest monocyte-cluster shifts. Classical monocytes therefore remained the most defensible downstream focus, while T-cell contributions should be tested more directly in larger single-cell cohorts. At the same time, pseudobulk results in classical monocytes suggested that the adverse-outcome-related state is not equivalent to simply “more inflammation,” but is accompanied by insufficient interferon support, persistent activation of inflammatory and coagulation programs, and metabolic impairment (35). These findings should still be understood as an association-based state description rather than a causal explanation of the endpoint.

Third, sensitivity and null analyses strengthened confidence in the axis from another angle. Regardless of whether recovery-related and failure-related gene-set sizes were adjusted from 10 to 30, the opposite disease-course trajectories in the discovery cohort, disease-state separation in external cohorts, and host heterogeneity stratification in GSE65682 (36) remained stable, whereas 28-day mortality stratification consistently lacked robustness. Supplementary Figure 2 is an effect-size and rank-correlation robustness figure rather than a repeated time-course trajectory plot. The new random-gene null analysis further indicated that the transferred score variance in external cohorts was far larger than expected from random matched gene sets, and the non-axis high-versus-low expression effects were strongly concordant across independent cohorts. Together, these results indicate that the central narrative of this study is not driven accidentally by a particular threshold or a few individual genes, but corresponds to a relatively stable host-state signal.

Fourth, further identification of internal program branches within classical monocytes gave the state framework higher resolution. Although small, the interferon-activated subpopulation showed the most prominent adverse-outcome-related axis displacement in most estimable leave-one-donor-out iterations, suggesting that the host’s capacity to rapidly establish an emergency interferon program may be a stratification clue worthy of further testing. The implication of the small sample size is important: with only seven donors and two non-survivors in GSE167363, substate-specific estimates are vulnerable to donor composition, sparse cell coverage, and endpoint imbalance. These results are therefore best treated as a prioritized validation target rather than a decisive branch that can already be concluded.

Fifth, monocyte-related communication analysis added an interaction-level layer to the findings. Monocytes not only displayed recovery-failure-related program differences internally, but also showed candidate interaction changes while transmitting key signals to T cells and platelets. These results are still insufficient to support causal conclusions about network remodeling, but they provide priority leads for future experimental validation centered on the monocyte-T cell-platelet axis.

Sixth, the external single-cell cohort suggests that different adverse endpoints are not completely homogeneous (37). Whereas the mortality-related cohort showed a more typical pattern of “low interferon plus heightened inflammation,” the acute respiratory distress syndrome-related cohort was closer to a branch marked by “upregulated interferon with reduced oxidative phosphorylation.” Importantly, this cohort did not reproduce the direction seen in the mortality cohort and therefore cannot be regarded as external validation for the same endpoint in the same direction. The value of GSE151263 lies mainly in supplementing our understanding of heterogeneity rather than replacing strict replication of the mortality endpoint.

The strengths of this study include its use of publicly available multi-cohort data, prespecified direction-based gene-selection rules, direct transfer of the discovery genes without retraining in external cohorts, mixed-effects modeling of all discovery time points, sensitivity analysis across gene-set sizes, random-gene null testing, cross-cohort non-axis transcriptomic concordance analysis, donor-level pseudobulk analysis to reduce single-cell pseudoreplication, original-cluster screening of T-cell heterogeneity, and leave-one-donor-out assessment for the small single-cell cohort. These features improve transparency and reproducibility compared with an analysis based only on single DEG lists or optimized classification performance.

In translational terms, the recovery-failure axis should not yet be used for direct single-time-point clinical risk judgment. Its near-term value is as a research framework for patient-state stratification, therapeutic-window identification, and testable hypothesis generation in sepsis (38, 39). A concrete prospective validation roadmap would include a multicenter longitudinal whole-blood cohort with matched clinical severity scores, source-of-infection data, treatment variables, and 28-day or longer-term outcomes. As a practical planning target, a bulk-transcriptomic validation cohort of approximately 150–200 patients, ideally enriched to include at least 40–60 non-survivors or other adverse-outcome cases, would allow more stable trajectory modeling and external calibration; final sample size should be determined by formal power calculations using pilot variance estimates. Sampling should begin at emergency department or ICU enrollment and include early 0–6 h sampling, 24 h, 72 h, day 5 to day 7, and a recovery or convalescent time point when feasible. A nested single-cell substudy of approximately 40–60 patients, enriched for at least 15–20 adverse-outcome cases, would be a reasonable starting design to test whether classical-monocyte and IFN-activated substate signals are reproducible. In particular, future work could examine emergency interferon programs in monocytes, maintenance of immunometabolism, and their coordination with T cells and platelets through in vitro interferon stimulation experiments, rescue interventions in sepsis-like animal models, and matched longitudinal clinical samples.

This study still has several limitations. First, all conclusions are derived from secondary analyses of public datasets and lack validation in prospective clinical cohorts and functional experiments. Second, the discovery-gene selection used day 5 versus day 1 contrasts as the main trajectory anchor. Although all day 1 to day 5 measurements were later incorporated in mixed-effects modeling, more flexible trajectory approaches, such as splines, latent class mixed models, or time-series clustering, could better use intermediate time points in future work. Third, the current cohort-wise z-score implementation limits immediate use for new single patients and prevents interpretation as a universal absolute cutoff; prospective deployment would require locked reference normalization, calibration samples, or rank-based single-sample scoring. Fourth, although the new random-gene null and non-axis concordance analyses support validity as a host-state coordinate, they still do not prove clinical utility or prospective actionability. Fifth, GSE167363 included only seven donors, of whom only two were non-survivors. Although pseudobulk analyses used the donor-time point-cell state as the statistical unit and were supplemented by leave-one-donor-out assessment, statistical power for substate refinement, T-cell subcluster screening, and cell-communication analysis remained limited. In particular, some T-cell clusters and the interferon-activated monocyte substate had sparse donor coverage, so these results should be regarded as exploratory evidence rather than definitive causal proof. Sixth, the day 1 to day 5 framework of the whole-blood longitudinal cohort does not correspond one-to-one with the 0- and 6-h framework of the single-cell cohort; this study achieved state anchoring across time scales rather than strict time-point-matched validation, and future prospective work should collect paired bulk and single-cell samples at aligned early and later time points. Seventh, bedside severity annotations were incomplete in public cohorts, and directly comparable SOFA annotations were unavailable. Eighth, the endpoint of the external single-cell cohort GSE151263 was not death but sepsis with acute respiratory distress syndrome; accordingly, its most appropriate role is to supplement heterogeneity rather than serve as strict directional replication.

## 5 Conclusion

Using public multi-cohort transcriptomic and single-cell data, we constructed and evaluated a sepsis recovery-failure axis that reflects host recovery tendency. The axis was jointly defined by multidimensional dynamic evidence, was transferable as a host-state stratification signal at the whole-blood level, exceeded matched random-gene expectations in external cohorts, and identified cross-cohort non-axis transcriptomic structure among high-score patients. At the single-cell level, the axis achieved cellular anchoring centered on classical monocytes, and additional original-cluster screening did not identify a robust hidden T-cell cluster that superseded monocyte anchoring. Its principal associated features included insufficient interferon support, persistent activation of inflammatory and coagulation programs, and metabolic impairment, while also suggesting monocyte-T cell-platelet interactions worthy of further validation. The recovery-failure axis showed only limited correlation with conventional APACHE II or SAPS II scores and remained robust across gene-set size settings, mixed-effects modeling, random-gene null analysis, and donor leave-one-out analyses. It is best interpreted as a host-state coordinate complementary to bedside scoring, not as a ready-to-use absolute clinical cutoff.

## Data Availability

The datasets analyzed in this study are publicly available in the NCBI Gene Expression Omnibus (GEO) repository (https://www.ncbi.nlm.nih.gov/geo/) under accession numbers GSE54514, GSE57065, GSE65682, GSE167363, and GSE151263. Further inquiries can be directed to the corresponding author.
