# Supplementary material for: Construction of a sepsis recovery-failure axis and single-cell anchoring using public multi-cohort whole-blood and single-cell transcriptomic data
Source: Front Med (Lausanne). 2026 Aug 6;13:1861140. doi: 10.3389/fmed.2026.1861140 (PMC13490106; doi:10.3389/fmed.2026.1861140)

Bulk validity analyses for the recovery-failure score

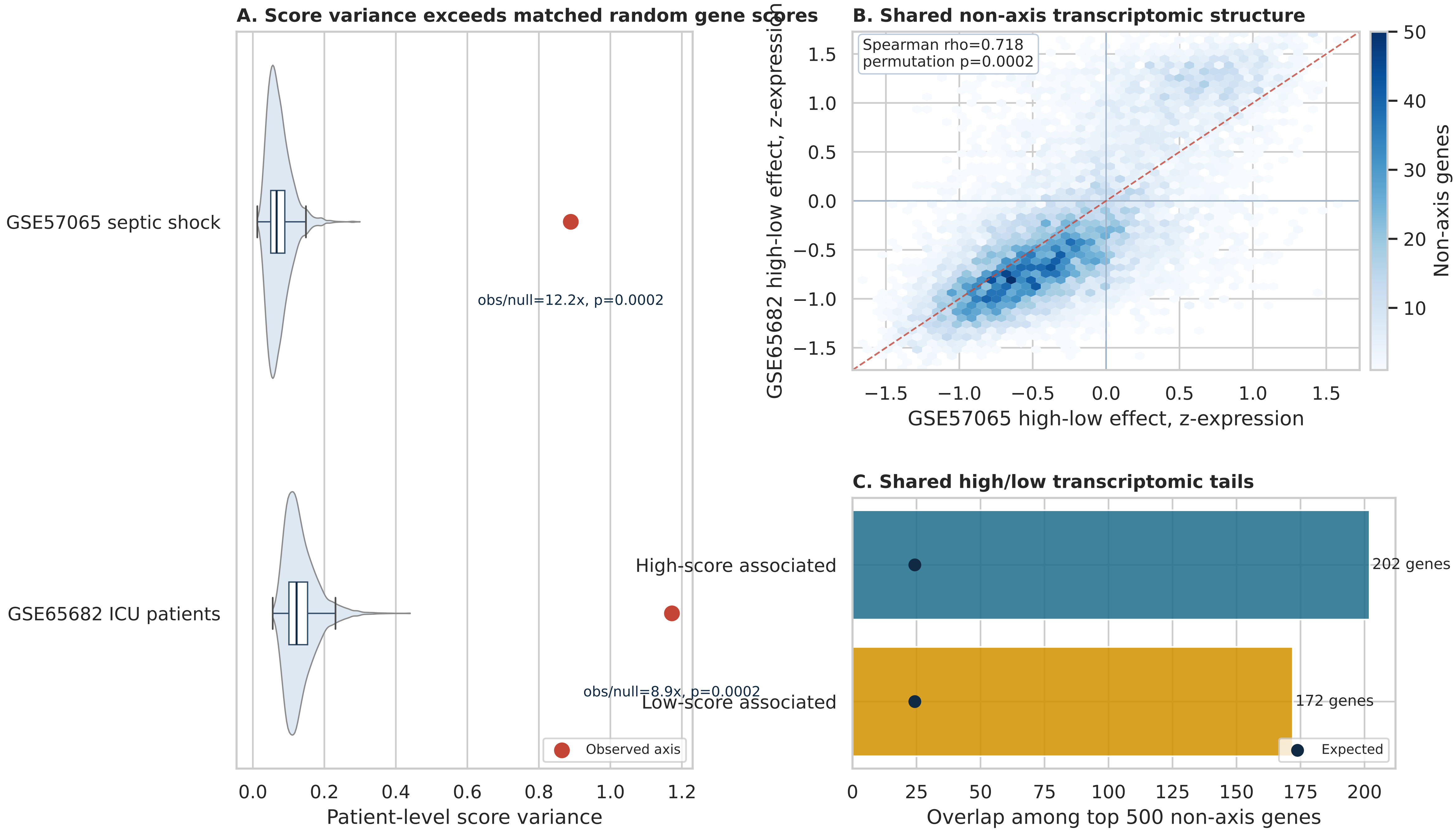

Supplement: Supplementary file 1 [file Image_1.pdf]
