# Supplementary material for: Construction of a sepsis recovery-failure axis and single-cell anchoring using public multi-cohort whole-blood and single-cell transcriptomic data
Source: Front Med (Lausanne). 2026 Aug 6;13:1861140. doi: 10.3389/fmed.2026.1861140 (PMC13490106; doi:10.3389/fmed.2026.1861140)
Supplement: Supplementary file 3 [file Supplementary_file_1.docx]

**Supplementary Figure S1 Association between the Recovery-Failure Axis and Published Clinical Severity Scores**

This figure illustrates the relationship between the recovery-failure axis and traditional bedside severity scores. The top left shows the scatter plot of Day 1 recovery-failure scores versus APACHE II in GSE54514; the top right shows the longitudinal trajectories under different APACHE II strata for patients with paired Day 1 and Day 5 samples; the bottom left shows the short-term changes at 0, 24, and 48 hours in high and low SAPS II groups in GSE57065. Overall, the results indicate that this axis has only limited correlation with APACHE II or SAPS II, further supporting its role as a host state indicator complementary to conventional severity scores rather than a simple substitute.

**Supplementary Figure S2 Robustness of the Recovery-Failure Axis to Gene Set Size Selection**

This figure shows a sensitivity analysis of the Recovery-Failure axis under different gene set size settings. The top left heatmap summarizes the direction and significance of key comparisons at 10, 15, 20, 25, and 30 recovery/failure genes; the top right shows the high correlation of alternative scores with the standard 20-gene score across three sets of bulk cohorts; the bottom left shows the stable pattern of core conclusion effect sizes as gene set size varies; and the bottom right provides an overall interpretation. This figure demonstrates that the main conclusions of this study are not driven by a single threshold or a few incidental genes, but correspond to a relatively stable host state signal.

**Supplementary Figure S3: All time points mixed-effects model and neutrophil proportion adjustment support the robustness of bulk trajectory bifurcation.**

This figure is used to illustrate that the trajectory differences observed in the discovery cohort are not driven solely by Day 1 and Day 5. The top row shows patient-level trajectories and mixed-effects model fits for all repeated measurements from Day 1 to Day 5 in GSE54514; the right panel further applies residual adjustment for neutrophil proportions provided by the public metadata. The bottom row summarizes fixed-effect estimates without adjustment, with neutrophil proportion adjustment, and further adjustment including APACHE II. The results show that the time × outcome interaction remains stable across different model settings, while neutrophil proportion and APACHE II have limited main effects, supporting the robustness of the bulk trajectory bifurcation described along the recovery-failure axis.

**Supplementary Figure S4 Single-cell leave-one-donor-out analysis supports the robustness of classical monocyte anchoring and defines the evidential boundaries for interferon-activated subtypes.**

This figure is used to supplementarily illustrate the robustness and limitations of the GSE167363 single-cell results at the donor level. The top left shows the donor-level recovery-failure axis means for major immune states at the 6-hour time point; the top right shows the stability of major immune state rankings by absolute axis values after leave-one-donor-out, with classical monocytes remaining first in all repetitions. The bottom left shows differences in monocyte subtypes along the non-survivor–survivor axis after leave-one-donor-out, indicating that the interferon-activated subtype shifts most noticeably in most estimable repetitions, but becomes unestimable after removing NSES due to the absence of this subtype in the remaining non-survivors. The bottom right shows subtype cell coverage for each donor and time point, providing a transparent depiction of the evid
